# Effects of Soil Temperature, Flooding, and Organic Matter Addition on N_2_O Emissions from a Soil of Hongze Lake Wetland, China

**DOI:** 10.1155/2014/272684

**Published:** 2014-07-14

**Authors:** Yan Lu, Hongwen Xu

**Affiliations:** ^1^School of Urban and Environmental Science, Huaiyin Normal University, Huai'an 223300, China; ^2^Jiangsu Key Laboratory for Eco-Agricultural Biotechnology around Hongze Lake, Huai'an 223300, China

## Abstract

The objectives of this study were to test the effects of soil temperature, flooding, and raw organic matter input on N_2_O emissions in a soil sampled at Hongze Lake wetland, Jiangsu Province, China. The treatments studied were—peat soil (I), peat soil under flooding (II), peat soil plus raw organic matter (III), and peat soil under flooding plus organic matter. These four treatments were incubated at 20°C and 35°C. The result showed that temperature increase could enhance N_2_O emissions rate and cumulative emissions significantly; moreover, the flooded soil with external organic matter inputs showed the lowest cumulative rise in N_2_O emissions due to temperature increment. Flooding might inhibit soil N_2_O emissions, and the inhibition was more pronounced after organic matter addition to the original soil. Conversely, organic matter input explained lower cumulative N_2_O emissions under flooding. Our results suggest that complex interactions between flooding and other environmental factors might appear in soil N_2_O emissions. Further studies are needed to understand potential synergies or antagonisms between environmental factors that control N_2_O emissions in wetland soils.

## 1. Introduction

Climate change has been a major issue within the twenty-first century. The main causes of global warming are the increase in carbon dioxide (CO_2_), methane (CH_4_), and nitrous oxide (N_2_O). Concentrations of N_2_O in the atmosphere are lower than those of either CH_4_ or CO_2_; however, on a per mole basis, N_2_O has demonstrated a higher ability to disrupt the radiation balance than the other two gases aforementioned. Thus, N_2_O has attracted much attention because it is a potent greenhouse gas with long atmospheric lifetimes, and it is involved in ozone depletion as well [1, 2]. Measurements of N_2_O emissions have been increasing steadily. Until now, however, the vast majority of studies have focused on N_2_O emissions from agroecosystem, as affected by temperature, soil type, crop growth, and management practices [3–5]. Therefore, there is much uncertainty surrounding the effect of flooding or the joint effects of several factors, for example, flooding and temperature changes.

Changes in land use patterns will cause alterations in the soil organic matter status and the soil nitrogen mineralization rates and therefore will also influence N_2_O emissions. In addition, land use changes may also affect transfer and diffusion of N_2_O in the soil through reorganization of the soil structure. Wetlands have been identified as major landforms regulating greenhouse gases dynamics [6]. CO_2_ and CH_4_ emissions from wetland system have been reported by numerous studies [7, 8]. However, research conducted on N_2_O emission from the wetland soil has relatively fewer reports. The predominant factors affecting N_2_O production and emission include soil temperature, soil moisture, exogenous organic matter inputs, and soil organic matter content [9–11]. They could have influence on microorganisms producing and consuming N_2_O from wetland soils [12]. Soil temperature directly impacts production and consumption of N_2_O though microorganism's activity, soil aeration, substrate availability, and redistribution. Soil moisture is a key determinant of the microbial processes [13]. Organic matter fractions have been also found to enhance N_2_O emissions as they supply substrates for nitrification and denitrification and augment microbial O_2_ consumption [14].

The interactions among soil temperature, moisture, and raw organic matter addition on N_2_O emissions are not well known and the gaps about the joint effects of these factors may increase the uncertainty in estimating global emissions budgets. Wetland soil from Hongze Lake was incubated under different temperature, moisture, and exogenous organic matter input. The objective was to analyze the interaction of soil temperature, flooding, and row organic matter inputs on N_2_O losses. Knowledge about the nitrogen loss process of the wetland ecological system may provide the baseline data to undertake countermeasures allowing reductions of greenhouse gas emissions.

## 2. Material and Methods

### 2.1. Site and Soil Characteristics

Hongze Lake is located in the northwest region of Jiangsu Province (33°06′ N~33°40′ N, 118°10′ E~118°55′ E), and it is the fourth largest freshwater lake in China. The lake surface area covers 1597 km^2^, the water level is 12.5 m, and the average depth is 1.9 m. The lake region has a monsoon climate with four distinct seasons. The average annual rainfall is 925.5 mm, mostly concentrated in the rainy season from June to September. The lake and the surrounding area are representative of inland wetlands in China. Wetlands are widely distributed around the lake; several wetland types have been described and the three most common are estuarine, floodplain, and out of the lake wetland.

Estuary wetlands located in the west coast of the Hongze Lake have been sampled in November 2012. Soil samples were collected at 0–20 cm depth using the multipoint hybrid method and taken to the laboratory in field-moist condition. Soil samples were divided into two subsamples, after manually removing visible plant roots and rocks. A subsample was put in the refrigerator (4°C) for incubation after sieving with 2 mm sieve, and the second subsample was dried and fine grinded for chemical analysis.

Basic physical and chemical properties of the tested soil are shown in Table 1. This soil was rich in organic matter with a total carbon content of 62.11 g*·*kg^−1^ and a total nitrogen content of 3.96 g*·*kg^−1^. Available nitrogen was as high as 382.81 mg*·*kg^−1^. Ammonia nitrogen (26.68 mg*·*kg^−1^) was approximately four times greater than nitrate nitrogen (6.55 mg*·*kg^−1^). Bulk density was 0.87 g*·*cm^−3^.

### 2.2. Incubation Experiment

An incubation experiment was setup to study NO_2_ emissions under four treatments and each treatment was incubated at two different temperatures. Treatments were as follows: natural peat soil (I), peat soil under flooding (II), peat soil plus raw organic matter (III), and peat soil under flooding plus organic matter (IV). Three replications per treatments were analyzed. To assess the effect of temperature on N_2_O treatments each replicate sample was incubated at 35°C (high temperature or treatment A) and at 20°C (room temperature or treatment B). At the same time, two blank tests were performed as a control treatment.

Soil sampled in the field was preincubated at room temperature for three days. Afterwards, 40 g of preincubated soil was enclosed in 1000 mL incubation bottles. Incubations were performed in 24 bottles (3 replicates × 4 different soil treatments × 2 temperatures). In the flooding treatment, 40 mL distilled water was added to the 40 g dry soil (soil was submerged by a 0.5 cm water column). In the treatment with exogenous organic matter addition, 1 g dried litter of* Phragmites communis* was grinded and mixed with the soil samples and added into each bottle. Bottles were sealed and maintained at constant temperatures of 20°C (treatment B) and 35°C (treatment A). Therefore, treatments incubated at high temperatures are labeled as AI, AII, AIII, and AIV, whereas labels for those incubated at low temperatures are BI, BII, BIII, and BIV.

### 2.3. N_2_O Analysis

Measures of N_2_O fluxes were a total of 12 times at the 2nd, 3rd, 5th, 7th, 9th, 11th, 13th, 16th, 19th, 22nd, 25th, and 28th days after incubation started. At each sampling date, 30 mL of gases was pumped from each incubation bottle. After then each bottle was open for half an hour to ensure that the gas in the bottle was equilibrated with that in the air and then the incubation bottles were again sealed. Gas chromatograph was used to measure the concentration of N_2_O in the samples. Emission rates were expressed as g*·*kg^−1^ *·*h^−1^ of N_2_O.

### 2.4. Data Analysis

The data set was analyzed by one-way analysis of variance (ANOVA) followed by Duncan's test at 0.05 level to compare the mean of N_2_O of each treatment. The SPSS 16.0 for Windows was used.

## 3. Results

### 3.1. Effect of Temperature on N_2_O Emissions

**Figure 1:**
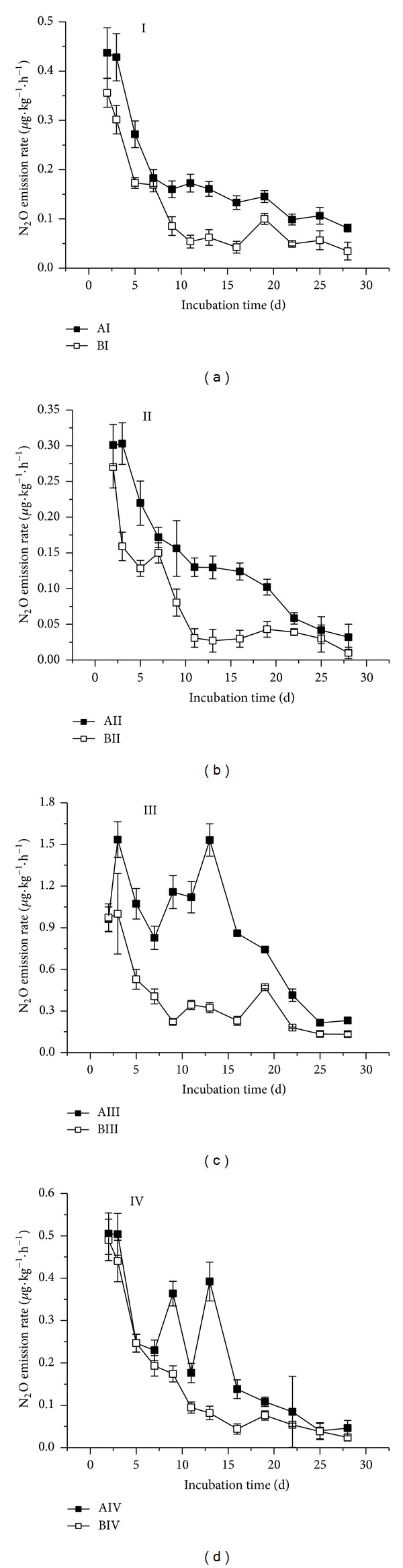
N_2_O emission rate of the treatments studied. (Treatments notations are as follows: A: incubation at 35°C; B: incubation at 20°C; I: peat soil; II: peat soil under flooding; III: peat soil plus raw organic matter; IV: peat soil under flooding plus organic matter).

**Figure 2:**
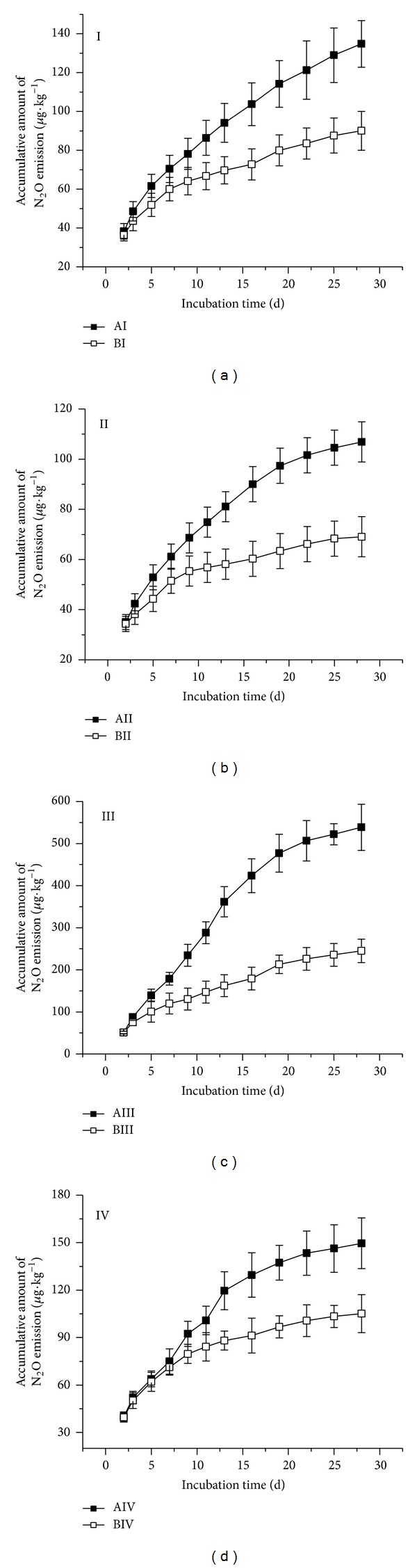
Cumulative amount of N_2_O emission of the treatments studied. (Treatments notations are as follows: A: incubation at 35°C; B: incubation at 20°C; I: peat soil; II: peat soil under flooding; III: peat soil plus raw organic matter; IV: peat soil under flooding plus organic matter).

It was found that all of the four treatments studied exhibited higher N_2_O emissions rates at 35°C than at 20°C. The highest N_2_O emissions rates occurred during the first periods of incubation and then gradually decreased (Figure 1). Moreover, complex changes of emission rates were noticed after organic matter was added to the wetland incubated at high temperature (Figure 1, AIV). Higher cumulative N_2_O emissions were observed under high temperature as well (Figure 2, BIV). N_2_O emission in treatment AI was 49.67% higher than in BI, AII was 54.78% higher than BII, AIII was 119.81% higher than BIII, and AIV was 42.33% higher than that of BIV. Moreover, highly significant differences were found between treatments AI and AIII (high temperature and peat soil versus peat soil plus exogenous organics matter) and also between BI and BIII (room temperature and peat soil plus exogenous organic matter) (*n* = 3, *P* < 0.05 or *P* < 0.01).

It could be deduced that increasing incubation temperature increased N_2_O emissions. The maximum accumulative increase in N_2_O emissions was for the peat soil added with raw organic matter, whereas the minimum accumulative was for the flooded peat soil added with raw organic matter.

### 3.2. Effect of Moisture Conditions on N_2_O Emissions

Both N_2_O emission rate and cumulative amount were lower in the flooding than in the unflooding treatment whether organic matter was added or no under high and room temperature conditions (Figures 1 and 2). N_2_O emissions in AII were 20.72% lower than in AI; AIV emissions decreased by 72.23% with respect to AIII; meanwhile, BII was 23.34% lower than BI and BIV declined by 57.11% compared to BIII. Significant differences were found between AI and AII, AIII and AIV, BI and BII, and BIII and BIV (*n* = 3, *P* < 0.05). These results show that irrespective of incubation temperature, flooding inhibits soil N_2_O emissions; moreover, the decline of N_2_O emissions under flooding was more obvious in soils plus organic matter.

### 3.3. Effect of Organic Matter Input on N_2_O Emissions

Further additions of organic matter to the studied peat soils increased soil N_2_O emission rates in the flooded and the unflooded treatments at the two levels of temperature studied (Figure 1). Moreover, sharp fluctuations of soil N_2_O emission along the incubation period occurred in soils added with organic matter under high temperature (AIII and AIV treatments). Organic matter additions clearly amplified cumulative amount of N_2_O emissions (Figure 2). N_2_O emissions in AIII were 299.62% higher than in AI, whereas those of AIV were 39.98% higher than those of AII. Also BIII emissions were 172.11% higher than in BI, and BIV were 52.23% higher than in BII. Very significant differences have been shown between AI and AIII (high temperature and peat soil versus peat soil plus organic matter), between AII and AII (high temperature and flooded soil versus flooded soil plus organic matter), and also between BI and BIII and BII and BIV (*n* = 3, *P* < 0.01). It was shown that the effect of exogenous organic matter input of increasing N_2_O emissions was relatively much lower under flooding conditions, compared to normal unflooding conditions.

## 4. Discussion

Soil temperature has a great influence on N_2_O formation through the biological processes of nitrification and denitrification. Optimum temperatures for nitrifying bacterial activities are in the range from 15 to 35°C, and nitrification will be inhibited at below 5°C or above 40°C. Optimum temperatures for denitrifying microorganism are from 30 to 67°C [15]. Soil temperature was an important factor affecting N_2_O emission as well. N_2_O emission rate was shown to be nearly synchronized with surface soil temperature, and the temperature variation was the main driver of diurnal and seasonal changes in N_2_O emissions [16]. In our wetland soil, N_2_O emission increased with increasing temperature, which was consistent with previous reports on forest soils [10, 17]. This result could be ascribed to the fact that rates of enzymatic reactions would increase with temperature when other environmental factors are not restrictive [18]. Moreover, temperature might regulate soil denitrification both directly and indirectly, and it could promote the activity of soil nitrifying and denitrifying microorganism and hence enhance N_2_O emission rate and cumulative emissions [19–22].

N_2_O production and emission may be influenced by soil moisture, through its effects on soil aeration status, reduction-oxidation conditions, microbial activities, and N_2_O diffusion into atmosphere. Feng and Yin [23] found that more N_2_O was produced at 45%–75% water filled pore space. When it was below the saturated water content, soil N_2_O emission increased with the increase of soil moisture, and nitrification was accredited as the basic source of N_2_O. However, when it was above the saturated water content, the denitrification process was the main source of N_2_O. With increasing soil moisture content above this threshold, the proportion of denitrifying N_2_ increased gradually and N_2_O emissions gradually weakened. Results from N_2_O emission observed in the rice and wheat crop rotation system in east China showed that if soil moisture content was less than 415 g*·*kg^−1^, then soil N_2_O emissions increased linearly with increasing soil moisture content; however, when the soil moisture content was more than 415 g*·*kg^−1^, N_2_O emissions reduced with the increase of soil moisture content [24], indicating much higher N_2_ loss under saturated soil water condition. Therefore, the production of N_2_O was not positively related to soil moisture under high moisture condition. Soil moisture content was also a key determinant of soil microbial activity [25], and it had impact on the diffusion of N_2_O produced during denitrification from microbial air into the surrounding environment [26]. Consequently, we deduced that both nitrifier-denitrifier microorganisms and coupled nitrification-denitrification processes contributed to the elevated N_2_O emission in wetland soil [27].

Organic matter input was thought to be an important regulator of N_2_O emissions, because organic matter on the soil surface could influence nitrification and denitrification reactions resulting from N mobilization and immobilization [28]. Toma and Hatano found huge N_2_O emissions when accepting low C/N ratio residues, mainly because these residues tended to break up [29]. Liu et al. reported that wheat straw incorporation increased N_2_O emissions, while the incorporation of maize straw had no impact on the emissions [30]. Millar et al. observed greater N_2_O emissions in areas cultivated with corn-wheat due to high amount of N applied to the corn field [31]. Therefore similar to our study, other results have shown that organic matter input enhanced soil N_2_O emission rate and cumulative emissions. The contribution of the organic matter could be mainly explained by the mineralization of litter which supplied substrates and thus promoted the production of N_2_O. In opposite, results about the negative role of organic matter on N_2_O emissions have also been reported [32]. Moreover, Tang et al. found that there were no significant impacts of organic matter on soil N_2_O emissions in a subtropical and two tropical forests [33–35], which underlies the potential adverse effects of organic matter. Consequently, we hypothesize that the net integrated effect of organic matter on N_2_O emission was the result of the counterbalance between the positive and negative feedbacks arising from interactions with other factors.

## 5. Conclusions

Both temperature rise and exogenous organic matter inputs increased N_2_O emission rates and cumulative amount from a wetland soil. The flooded soil with external organic matter inputs showed the lowest cumulative rise in N_2_O emissions as the temperature increased. Irregular changes of N_2_O emission along the incubation period were found in soils added with organic matter under high temperature condition. Soil N_2_O emissions could be inhibited by flooding; however, complex internal connections and cooperative or antagonistic actions between flooding and other environmental factors might appear in soil N_2_O emissions. Thus, it is recommended to conduct further studies for assessing the way through which other environmental factors affect soil N_2_O emissions of wetland ecosystem.

## Figures and Tables

**Table 1:** Basic physical and chemical properties of studied soils.

Total carbon (g*·*kg^−1^)	Total nitrogen (g*·*kg^−1^)	Ammonia nitrogen (mg*·*kg^−1^)	Nitrate nitrogen (mg*·*kg^−1^)	Available nitrogen (mg*·*kg^−1^)	Bulk density (g*·*cm^−3^)
62.11	3.96	26.68	6.55	382.81	0.87
